# Preparedness is key in the face of avian influenza uncertainty

**DOI:** 10.1016/j.nmni.2024.101505

**Published:** 2024-10-10

**Authors:** Abdullahi Tunde Aborode, Ridwan Olamilekan Adesola, Godfred Yawson Scott, Paulina Morales Ruiz

**Affiliations:** Healthy Africans Platform, Research and Development, Ibadan, Nigeria; Department of Veterinary Medicine, Faculty of Veterinary Medicine, University of Ibadan, Ibadan, Nigeria; Department of Medical Diagnostics, Kwame Nkrumah University of Science and Technology, Kumasi, Ghana; Access-To-Medicines Research Centre, Mexico

**Keywords:** Avian influenza A, H5N2, Preparedness, Mexico

Dear Editor,

Avian influenza poses significant public health risks due to its potential for zoonotic transmission. Influenza A viruses can be categorized as avian, swine, or other animal influenza viruses based on their initial host. Influenza viruses that affect animals can also infect people and are mostly contracted via direct contact with diseased animals or contaminated surroundings (Fig. 1) [1]. Consequently, the occurrence of isolated human cases is expected. Previously, there have been recorded examples of humans being infected with several H5 subtypes, including A(H5N1), A(H5N6), and A(H5N8) viruses [1].

**Fig. 1:**
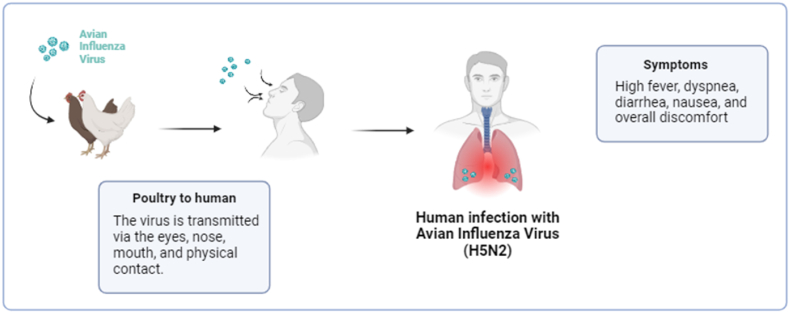
Shows the transmission of avian influenza virus (H5N2).

Human infections with avian influenza viruses can result in varying degrees of upper respiratory tract infections, ranging from mild to severe, and have the potential to be lethal. Additionally, cases of conjunctivitis, gastrointestinal problems, encephalitis, and encephalopathy have been documented. Diagnostic laboratory tests are necessary to identify and confirm cases of influenza in humans. The World Health Organization (WHO) advises procedures for the detection of zoonotic influenza utilizing molecular technologies, such as reverse transcription polymerase chain reaction (RT-PCR). No targeted vaccinations are available to prevent human infection with the influenza A(H5) virus [1].

The recent case of human H5N2 infection in Mexico, reported on May 23, 2024, emphasizes the urgent need for robust surveillance and response strategies. This study aims to discuss the case, response actions, and the broader implications for public health preparedness.

On May 23, 2024, the Mexico International Health Regulations National Focal Point reported to the Pan American Health Organization/World Health Organization (PAHO, WHO) a verified case of human infection with avian influenza A(H5N2) virus [1]. Notably, this person had no previous contact with poultry or other animals. The case had several coexisting medical problems, including chronic kidney disease, diabetes type 2, and long-standing systemic hypertension. On April 17, the individual experienced fever, dyspnea, diarrhoea, nausea, and overall discomfort. On April 24, the individual sought medical assistance, was admitted to the National Institute of Respiratory Diseases “Ismael Cosio Villegas” (INER), and passed away on the same day owing to complications related to their coexisting medical problems [1,2].

On April 24, the respiratory sample of the deceased was subjected to a Real-Time Polymerase Chain Reaction (RT-PCR) at the Instituto Nacional de Enfermedades Respiratorias (INER). It revealed the presence of an influenza A virus [2]. On May 8, the sample was sequenced at the Laboratory of Molecular Biology of Emerging Diseases Center for Research in Infectious Diseases (CIENI)at INER and tested positive for influenza A(H5N2) [2]. Subsequently, on May 20, the sample was received at the Institute of Epidemiological Diagnosis and Reference (InDRE) for further analysis. On May 22, InDRE's sequencing findings reported the presence of influenza subtype A (H5N2), confirming the earlier finding of CIENI [2]. The genetic analysis showed a 99 % similarity with the low pathogenic avian influenza (LPAI) A/chicken/Texcoco, México/CPA-01654/2024 (H5N2) strain identified in Texcoco, Mexico, in March 2024 [1]. While avian influenza viruses can infect humans through direct contact with infected animals or contaminated environments [1], isolated human cases are sporadic. A multidisciplinary team, including epidemiologists, infectious disease specialists, pulmonologists, microbiologists, and critical care experts, determined that the patient's death resulted from chronic health issues leading to multi-organ failure, not the H5N2 virus itself [2].

Seventeen individuals recognized as contacts of the deceased person were closely observed at the INER, and only one person reported experiencing a runny nose on April 28th and 29th [2]. Samples collected from these individuals were tested on May 27 and May 29, yielding negative results for influenza and SARS-CoV-2 [2]. Additionally, 12 individuals (seven showing symptoms and five not showing symptoms) living near the house of the person in question were tested using their pharyngeal exudate, nasopharyngeal swabs, and serum samples on May 28; none of the samples tested positive for SARS-CoV-2, influenza A, or influenza B, as verified by RT-PCR. As of June 15, the serological samples are still pending [1].

Following the identification of the case, national authorities implemented immediate actions endorsed by the WHO One Health strategy; they involved collaborative efforts between human health, epidemiology centres, and agricultural and environmental agencies that also surveilled animal health [2]. Moreover, local and national health authorities conducted surveillance of the patient's contacts and healthcare personnel who had previous interactions with the patient. Healthcare sectors also monitored and surveyed influenza-like respiratory illness (ILI) and severe acute respiratory illness (SARI) in nearby municipalities to study the patterns and trends of respiratory syndromes, identify transmission networks and determine risk variables in the city where the patient lived, as well as in neighbouring areas [2].

Based on the current evidence, with no more instances of human infection with A(H5N2) linked to this case and no human-human infections having been identified [2], the World Health Organization (WHO) evaluates the present risk of this virus to the general population as low [1,2].

Although the individual did not die from H5N2, detecting a previously unrecognized virus in humans is a critical finding. As uncertainty emerges about the potential of this virus to spread from human to human, the best course of action is to prepare. Therefore, revising and reinforcing the Mexican pandemic preparedness plan and global pandemic preparedness protocols is not just important but crucial in the face of this new threat.

The PAHO recommends structuring pandemic preparedness around five key areas: collaborative surveillance, safe and scalable care, access to countermeasures, emergency coordination, and community engagement [3]. Central to community engagement is the importance of effective risk communication. Experts must communicate with authorities and ensure that the public receives timely, relevant, and reliable information that addresses their needs and concerns. Keeping the community well-informed allows individuals to acknowledge the health risks they face and understand the actions they can take to protect their health and lives.

Communication should also highlight the One Health approach, emphasizing the interconnection between human, animal, and environmental health, and changes in the environment can impact animal and human health, leading to repercussions such as the emergence and spread of infectious diseases. Informing the community about these health interconnections will make them more conscious of their relationships with animals and the wider environment. This awareness can lead to more responsible behaviour and decision-making, ultimately helping to prevent the spread of infectious diseases and promoting overall community resilience to health threats. While Mexican authorities have already implemented a One Health approach to investigate the H5N2 case further, it is essential to strengthen this approach at various levels to enhance its effectiveness [2].

In Mexico, the 2009 H1N1 epidemic and the COVID-19 pandemic have demonstrated the importance of enhancing preparedness and response to pathogenic threats. Effective prevention is achieved through collaborative efforts, community engagement, and prompt actions [4,5]. As we face the uncertainty of a newly detected H5N2 virus in humans, improving preparedness capacities to address this and other emerging pathogens is vital.

The emergence of avian influenza A(H5N2) in a human patient in Mexico underscores the ongoing threat posed by zoonotic diseases and highlights the critical importance of preparedness. Despite the patient's death being attributed to underlying health conditions rather than the virus itself, the detection of H5N2 in humans necessitates heightened vigilance and readiness. Strengthening surveillance systems, adopting the One Health approach, and enhancing pandemic preparedness plans are vital steps in mitigating the risks associated with such pathogens. Effective risk communication and community engagement are essential to ensure public awareness and compliance with health measures. Continued research and international collaboration will be vital in addressing future outbreaks and protecting global public health. By reinforcing these strategies, we can better prepare for and respond to emerging zoonotic threats, ultimately safeguarding human, animal, and environmental health.

## CRediT authorship contribution statement

**Abdullahi Tunde Aborode:** Writing – review & editing, Writing – original draft, Data curation, Conceptualization. **Ridwan Olamilekan Adesola:** Writing – review & editing, Writing – original draft, Validation, Software, Data curation. **Godfred Yawson Scott:** Writing – review & editing, Writing – original draft, Visualization, Data curation. **Paulina Morales Ruiz:** Writing – review & editing, Writing – original draft, Validation, Data curation.

## Ethics approval and consent to participate

Not available.

## Consent for publication

Not applicable.

## Availability of data and material

The corresponding author has the right to share the data available in the manuscript.

## Competing interests

The authors declare that they have no competing interests.

## Funding

There is no funding for this study.

## Declaration of competing interest

The authors declare that they have no known competing financial interests or personal relationships that could have appeared to influence the work reported in this paper.
